# “There’s a lot of unknowns”: a thematic analysis of the experiences of young adults with cancer who died during a psychosocial intervention trial

**DOI:** 10.1186/s12904-025-01725-2

**Published:** 2025-04-09

**Authors:** Madeline H. Bono, Samantha Reese, Kristine Levonyan-Radloff, Kristine A. Donovan, Olle Jane Z. Sahler, Marie E. Barnett, Meredith Collins, Katie A. Devine

**Affiliations:** 1 Department of Pediatrics, Rutgers Cancer Institute of New Jersey, New Brunswick, NJ USA; 2 Department of Health Outcomes and Behavior, Moffitt Cancer Center, Tampa, FL USA; 3 Department of Psychiatry & Psychology, Mayo Clinic, Rochester, MN USA; 4 Division of Pediatric Hematology/Oncology, Golisano Children’s Hospital, University of Rochester School Medical Center, Rochester, NY USA; 5 Department of Psychiatry and Behavioral Science, Memorial Sloan Kettering Cancer Center, New York, NY USA

**Keywords:** Oncology, Cancer, Bright IDEAS, Young adults, Qualitative research, End-of-Life, Advanced care planning, Palliative

## Abstract

**Background:**

Young adults (YAs) with cancer of any stage face challenges and unmet needs across biopsychosocial domains. YAs who have rapidly declining health trajectories, or who enter end-of-life stage sooner-than-anticipated, merit greater understanding so their providers can prioritize patients’ needs and values during this vulnerable period. This analysis sought to explore the lived experiences and priorities of patients whose cancer progressed rapidly, or who entered end-of-life stage unexpectedly, by conducting a thematic analysis on transcripts generated by their participation in a randomized control trial (RCT) designed for YAs with newly diagnosed cancer.

**Methods:**

During a multisite RCT evaluating the efficacy of Bright IDEAS-YA, a 6-session psychosocial problem-solving intervention for YAs newly diagnosed with cancer, all Bright IDEAS-YA intervention sessions were recorded. Despite RCT eligibility criteria designating that participants should have a life-expectancy of 6 + months, some enrolled participants were unable to continue study participation due to death or rapid health decline. This analysis identified each participant who died during study participation or withdrew due to rapid health decline that had been enrolled into the intervention arm of the Bright IDEAS-YA RCT (*n* = 12). Individuals completed between 2 and 6 Bright IDEAS-YA sessions, which were transcribed verbatim for analysis (*n* = 60 transcripts). From December 2022 to March 2023, 10 researchers reviewed all available Bright IDEAS-YA transcripts and inductively co-developed a codebook on emergent transcript themes. Two researchers then co-coded all Bright IDEAS-YA transcripts, establishing a cut-off date of December 31, 2023 for participants eligible for subanalysis, once thematic saturation was determined.

**Results:**

Emergent themes included Cancer and Treatment, Mortality, Social Systems, Emotions, Work/Academics, Mental Health, Spirituality, COVID-19, Meaning Making, and Participation in Bright IDEAS-YA. Only a subset of themes reflected end-of-life challenges.

**Conclusions:**

YAs who approached end-of-life or saw unexpectedly fast declines in their healthcare trajectory showed many similarities to YAs outside of end-of-life contexts. Some, but not all, participants reflected on goals and challenges related to end-of-life during Bright IDEAs-YA participation. To ensure YAs are holistically understood, and that their priorities both prior to and during end-of-life are respected by their healthcare teams, it is important that providers collaborate with YA patients and introduce care strategies that elicit patients’ values and goals.

**Trial registration:**

ClinicalTrials.gov, #NCT04585269, October 14, 2020.

**Supplementary Information:**

The online version contains supplementary material available at 10.1186/s12904-025-01725-2.

## Background

For patients facing terminal illness, quality end-of-life care necessitates that patients’ and their families’ priorities are centered [1–3]. Literature examining the perspectives of patients with terminal illness illustrates that patients have needs related to agency, accepting changing health statuses, maintaining experienced normality, and having support of family and friends [1, 3–5]. For researchers seeking to represent the perspectives of individuals receiving end-of-life care, sample cohorts are typically comprised of individuals with advanced stage, life-limiting disease [3, 5, 6]. Life-limiting illnesses, however, do not always follow a predictable path, and for some individuals, the progression to advanced stage disease or death occurs unexpectedly [7, 8]. Thus, the standard pathway for recruitment in studies that capture patients’ perspectives at end-of-life often overlooks patients whose prognoses unexpectedly and/or rapidly decline.

Specifically looking at cancer as potentially life-limiting illness; young adults (YAs) with cancer–individuals between ages 18 and 40 – have been historically underrepresented in research, falling into a gap between pediatric and older adult oncology [9]. Due to exponential changes in socio-developmental needs and expectations for individuals moving through young adulthood – developing careers, making and fostering relationships, and building families – YAs with malignancy of any stage report more psychosocial needs in the areas of work, family, relational stress, finances, and identity development than other age cohorts [10–12]. For YAs with advanced stage cancer, qualitative explorations into these patients’ experiences indicate patients navigate increased levels of pain, fatigue, and existential or spiritual distress [13, 14]. These investigations face the same recruitment limitations described above, as it is difficult to recruit in a way that represents the perspectives of YAs whose malignancies progress rapidly and/or unexpectedly. For these patients, research has primarily been retrospective, or outcome-based [15, 16]. This has led to ongoing calls for improvement in understanding end-of-life care for YAs, through research that centers YA patient and family perspectives during the end-of-life period [15–17].

This manuscript reviews transcripts derived from a multisite randomized control trial (RCT) of a problem-solving skills training intervention for YAs recently diagnosed with cancer [18]. RCT eligibility criteria sought to exclude participants whose cancer was anticipated to lead to mortality within 6 months, but nonetheless, some participants died or were withdrawn due to health decline during RCT participation. This circumstance provided a unique opportunity to examine the lived experiences and priorities of people near end-of-life, whether or not they were aware of or acknowledged their proximity to end-of-life. By identifying these RCT participants post-hoc and qualitatively examining transcripts of their RCT participation, authors aimed to use thematic analysis to better illustrate the contemporaneous experience of YAs with cancer diagnoses that did, or would soon, rapidly progress to end-of-life.

## Methods

### Randomized control trial for Bright IDEAS-Young Adults

This sample was identified from participants enrolled in a multisite RCT examining the efficacy of Bright IDEAS-Young Adults (Bright IDEAS-YA), which was being conducted across three US cancer centers [18]. Bright IDEAS-YA is a manualized problem-solving skills training intervention, adapted for YAs, designed to help participants apply effective problem-solving strategies [18, 19]. This intervention is delivered by a Bright IDEAS-YA trainer/coach in six sessions over a 6–12-week period. These sessions include an introductory session where participants share their cancer story and learn the Bright IDEAS model^1^, four sessions where participants practice the model with self-generated goals or challenges, and a final session where participants complete a review of the Bright IDEAS-YA model and reflect on how to continue using the skills moving forward.

RCT recruitment began in February 2021 [18]. Inclusion criteria for this RCT were (1) recent initial diagnosis of any cancer (within 4 months), (2) receiving systemic cancer treatment, and (3) oncology team determination that patients had > 6 months life expectancy (i.e., the duration of the intervention and primary outcome measures [18]). Participants in the intervention arm of the RCT were expected to complete the manualized six Bright IDEAS-YA sessions with an assigned Bright IDEAS-YA trainer within 12 weeks of study recruitment [18]. Each session was held virtually via HIPAA-compliant telehealth platform and lasted up to one hour. All Bright IDEAS-YA sessions were audio-recorded with patient consent as a component of the Bright IDEAS-YA protocol [18].

### Study team

The research team involved in this analysis comprised an 11-person interdisciplinary team of researchers – with specialties in medicine, psychology, social work, and population science – at career stages that spanned from doctoral students to later career doctoral level researchers. All researchers have experience working in oncology and were involved in the larger Bright IDEAS-YA RCT.

### Data collection

#### Participants and transcripts

Per RCT inclusion criteria, all participants had received an initial cancer diagnosis within the past four months and were expected to live longer than six months, per their oncology care teams. Study team identified Bright IDEAS-YA participants who prematurely ended RCT participation due to death or being withdrawn due to severity of medical crises between February 2021 and December 2023. Only participants in the intervention arm of Bright IDEAS-YA (total 166 during this period) were reviewed by study team, as the Bright IDEAS-YA intervention transcripts were the data used for qualitative analysis. Fourteen (12%) of total participants in the intervention arm of Bright IDEAS-YA RCT died or were withdrawn due to medical crisis during the sample period. Of these 14 participants, 12 participants had transcribable Bright IDEAS-YA session recordings and two did not (one due to technical difficulties with recording and one due to participant having died prior to completing any Bright IDEAS-YA sessions). There were a total of 60 transcripts of Bright IDEAS-YA sessions from the 12 participants (i.e., 2–6 sessions per participant). For details on participant characteristics and the distribution of transcribed recordings, see Table 1.

**Table 1:** Participant characteristics

Number	Age at Time of Enrollment	Gender	Racial/Ethnic Background	Cancer Type	# of Bright IDEAS Audio Transcriptions	Time between Last Bright IDEAS Session and Death (months)
1	36	Woman	Asian, non-hispanic	Breast Cancer	6	12.7
2	20	Man	Asian, non-hispanic	Sarcoma	6	14.7
3	23	Woman	Other, hispanic	Sarcoma	6	6.1
4	20	Man	White, non-hispanic	Head and Neck Cancer	2	4.3
5	24	Woman	White, non-hispanic	Sarcoma	6	5.4
6	39	Man	Black, non-hispanic	Colorectal Cancer	4	1.2
7	39	Woman	White, non-hispanic	Sarcoma	2	N/A*
8	35	Woman	Asian, non-hispanic	Thoracic Carcinoma	6	1.6
9	32	Woman	White, non-hispanic	Skin Cancer	6	8.0
10	33	Woman	White, non-hispanic	Breast Cancer	4	15.2
11	31	Woman	Black, non-hispanic	Breast Cancer	6	10.2
12	36	Woman	White, non-hispanic	Myelodysplastic Syndrome	6	11.3

*Patient was withdrawn by oncology team

### Analysis

An exploratory thematic approach was used to develop a codebook to guide analysis of the transcript data [20].

#### Codebook development

Between December 2022 and March 2023, 10 research team members participated in biweekly meetings to develop a codebook to guide analysis. During this period, 34 Bright IDEAS-YA session transcripts of patients who had died or been withdrawn due to health decline were available to researchers (remaining eligible participants had not yet been identified). Researchers individually took notes during transcript readthroughs and identifying perceived themes [21]. During meetings, researchers reviewed their notes and co-developed a preliminary codebook based on convergent themes and subthemes identified between researchers [22]. The codebook was iterative in that, as further transcripts were reviewed throughout coding and researchers applied codes, redundant codes were collapsed and novel codes/subcodes were added as needed. Once coders had determined no substantial edits/additions were being made to codebook as new transcripts were being coded, thematic saturation was determined and December 30, 2023 was set as the cut-off date for participant eligibility [23]. The final codebook is available in Supplemental Materials.

#### Coding and analysis

Two researchers (MHB and SR) of the original 10-person codebook development team independently coded participant utterances from each full transcript using Dedoose software. Bright IDEAS-YA trainer utterances were not coded to ensure codes reflected participants’ experiences. Each excerpt could have as many codes as relevant applied. The two researchers met weekly to review coded transcripts and to resolve discrepancies between raters. Final codes were determined based on coder consensus. Once final codes were established, researchers exported each parent code from Dedoose and created aggregate summaries of excerpts for each code. Researchers then reviewed summaries together to finalize themes. During this phase, researchers used diagramming to make sense of thematic connections, aiding researchers in collapsing and refining themes cohesively [21].

## Results

The majority of participants were women (75%) and had a mean age of 30.67 years (standard deviation [SD] = 7.1 years; median = 32.5 years) at time of study enrollment. Cancer types included sarcoma (*n* = 4, 33%), breast cancer (*n* = 3, 25%), head and neck cancer (*n* = 1, 8%), colorectal cancer (*n* = 1, 8%), skin cancer (*n* = 1, 8%), thoracic carcinoma (*n* = 1, 8%), and myelodysplastic syndrome (*n* = 1, 8%). Mean time between last completed Bright IDEAS-YA session and participant death was 8.2 months (SD = 5.0 months; range = 1.2–15.2 months). Please refer to Table 1 for further participant details.

**Fig. 1:**
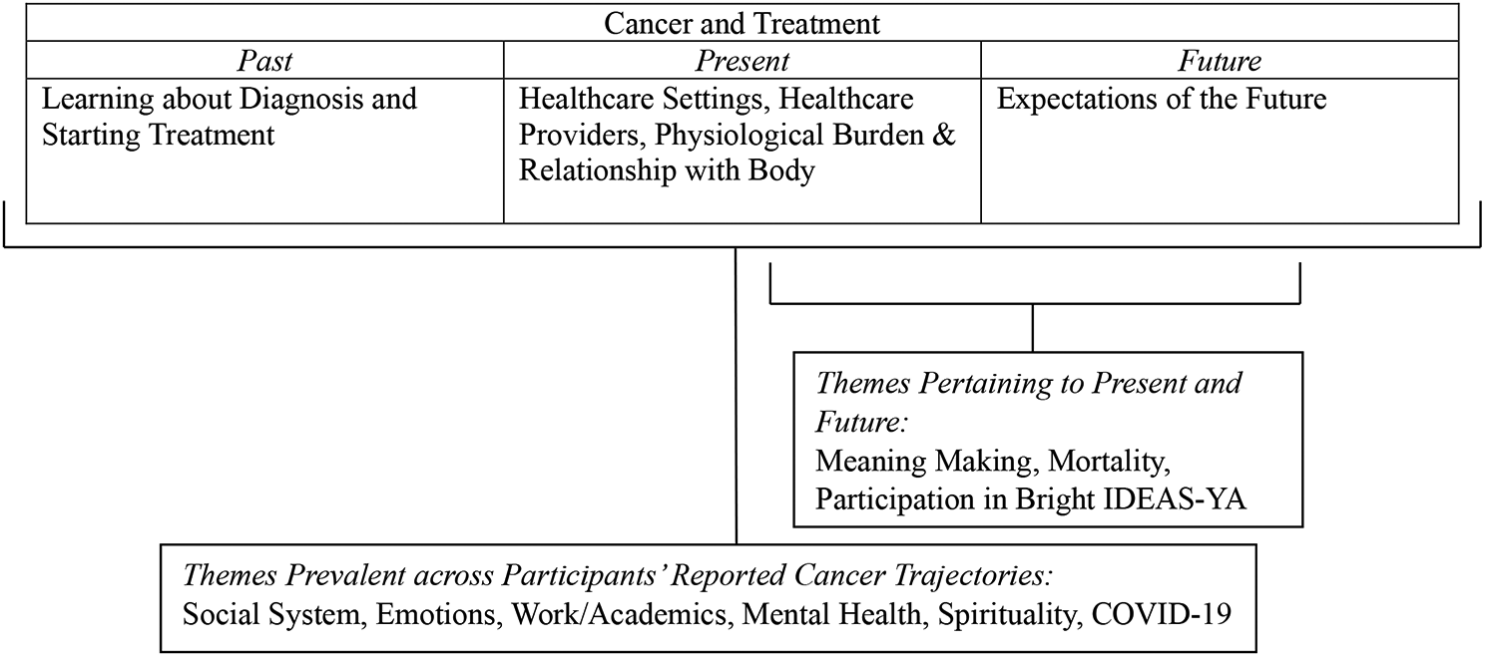
Visualization of Final Themes Drawn from Transcript Analysis

Emergent themes – comprising Cancer & Treatment, Mortality, Social System, Work/Academics, Mental Health, Spirituality, COVID-19, Meaning Making, Participation in Bright IDEAS-YA, and Emotions – are presented below. Participant quotes illustrating presented themes are included, following which participant ID numbers and age are listed. See Fig. 1 for a corresponding visualization created during diagramming.

### Cancer & treatment

Given the context of this analysis, participants’ cancer experiences comprised a central theme and framed other emergent themes.

#### Receiving diagnosis and starting treatment

During the first manualized Bright IDEAS-YA session, trainers encourage participants to review their experience receiving their cancer diagnosis and beginning treatment. Several participants (*n* = 5, 42%) expressed that their initial presenting symptoms felt innocuous: “*So it’s a really bizarre thing. All these tiny symptoms that you’d never think of doing anything turn out to be cancer.”(#4*,* age 20).* Participants frequently endorsed that their cancer diagnosis had been surprising to them. Another prevalent theme was time between symptom presentation, diagnosis, and treatment initiation felt rapid.

*“It was really fast*,* actually… I was there for two weeks. So like I went in for a cough at urgent care and then two weeks later*,* I was having my first chemo session.”(#8*,* age 35).*

#### Healthcare settings

Participants discussed clinical settings in the contexts of their diagnosis and treatment trajectory; decision-making about medical care (i.e. exploring second opinions through other hospital systems); their receipt of care; and resources provided through healthcare settings. Participants reviewed receiving care from primary care providers, specialty outpatient services, and emergency room (ER) visits, oncology services – through both their established care team and alternate care teams for second opinions – and inpatient hospitalization.

Several participants (*n* = 5, 42%) reflected at length on their experiences in inpatient care and three of these participants generated Bright IDEAS-YA goals to reduce distress associated with inpatient stays. Specific challenges endorsed included: loneliness; feeling isolated from support networks; limited access to coping strategies available at home; and discomfort in the hospital environment due to harsh lighting, sounds from monitors or other devices, and around-the-clock check-ins by medical staff that disturbed sleep and comfort.

*“It’s [inpatient care] a small room. It’s tan walls. It’s dealing with the brain fog and not being able to really focus and concentrate on anything…The first day you know is fine*,* but then from there on out it kind of just gets worse… I’ve been like just today kind of struggling with just the thought of doing that again.”(#7*,* age 39).*

Clinical care settings served as access points for other supportive resources (e.g. therapy referrals, cancer support groups, complementary and integrative oncology referrals). Participants explored supportive resources using (1) direct provider-patient communication and (2) and clinical care setting websites.

#### Healthcare providers

Participants’ healthcare providers included primary care providers, oncologists, ER staff, radiologists, nursing staff, embedded behavioral health providers (e.g. psychologists, social workers), nutritionists, and pastoral care. Patients viewed them as providing support by giving information about cancer trajectory and prognoses; encouraging treatment adherence; offering referrals and linkage to resources; and setting expectations for the impact of cancer treatment.

Provider feedback often affected patients’ decision-making, at least in part because of the relationship and shared goals: “*We have such a good team of doctors on my side*,* and I know that him saying that I should postpone my trip is really just for my own benefit and he’s right. At first*,* I was tempted…But*,* no*,* the most important thing is my health*,* which we’re working towards.” (#5*,* age 24).*

#### Physiological burden & relationship with body

Participants described how cancer and its treatments affected their physiological experiences and functioning. Participants reported being more aware of novel physical sensations and described worry that new sensations might be associated with worsening of their prognosis.

*“So my tumor’s on the left side*,* and this pain is kind of – I’ve been feeling it on both sides but more the right side. So I am just obviously terrified that it has spread*,* and I can’t imagine that it did*,* but at the same time*,* obviously that’s still on my mind.” (#5*,* age 24).*

Some participants (*n* = 4, 33%) expressed feeling reduced trust in their bodies and having more concerns that further physiological challenges might emerge, even beyond those associated with cancer.

Participants often reflected on changes they experienced in their physical capacity to engage in daily activities, including childcare, college, work, exercise, and hobbies. All participants (*n* = 12, 100%) sought to use Bright IDEAS-YA strategies to manage experiences associated with these changes. The most frequently identified Bright IDEAS-YA goals were related to movement for everyday functioning and exercise (*n* = 8, 67%). Participants regularly discussed adjusting routines and expectations of physical capacity: “*I think right now I’m just doing too much when I should be easing myself back into things*,* I guess. So I’m just like pushing myself too hard.” (#2*,* age 20).*

Participants also spoke about needing to adjust their diet around physiological changes and side-effects associated with treatment. Nausea, mucositis, dietary restrictions, and appetite changes impacted food choices and nutritional goals.

#### Expectations of the future

Many participants described uncertainty about their future and their prognosis (*n* = 10, 83%), which contributed to distress. Participants’ anxiety included not only worry about their overarching cancer trajectory – including potential worsening or recurrence – but also their fearful anticipation of the results of tests and scans. Some participants expressed confidence that they would survive their cancer.

*“I have come to the realization that stage four cancer is a pretty serious situation*,* so sometimes I get – when we’re going through testing*,* just conversations with doctors and so forth. But I have the mindset that we’ll beat it*,* and I’ll be here.” (#7*,* age 39).*

Others conveyed expectations that they would die of their cancer: *“I basically have triple-threat*,* plus on top of it being genetic… I’ll be lucky if I survive.” (#10*,* age 33).*

### Mortality

Ten out of 12 participants discussed mortality, at least referentially, during Bright IDEAS-YA sessions. Most participants who spoke about their own mortality (*n* = 6, 50%) first referred generally to the concept (e.g. mentioning others who have died of cancer), before linking this idea to themselves. Participants varied in how much abstraction they used to discuss their own potential death. Some participants (*n* = 4, 33%) spoke about their death as unlikely or hard to accept: *“One of the first things that I told everyone is*,* I needed them to not treat me like I just received a death sentence*,* because I don’t feel that way*,* and I’m not taking it that way.” (#11*,* age 31)*.

Some participants (*n* = 3, 25%) learned that their cancer was terminal over the course of their Bright IDEAS-YA participation. Participants who expected to die soon often stated that this informed their decision-making and impacted how they prioritized challenges and goals: *“Knowing that I only have like a certain timeframe… just I feel like some things are not worth chasing right now.” (#3*,* age 23)*.

Some participants (*n* = 5, 42%) reflected on their experiences discussing death with loved ones. Misalignment of expectations was identified as impeding communication. This was true both for participants who anticipated they would die – but whose loved ones expected more positive treatment outcomes – and for participants where the inverse was true. One participant who anticipated recovering from their cancer reflected that this dynamic with family members was frustrating: *“What I need is support and encouragement and positive words*,* and all of those things. And not for you to come visit and then to look at me like I’m dying*,* or like you feel sorry for me when that’s not the case.” (#11*,* age 31)*. Some participants (*n* = 2, 17%) whose perspectives on mortality aligned with loved ones described sharing in their grief together. Others (*n* = 2, 17%) reported that they and their loved ones were intentional about avoiding the topic: *“We’ve talked about it [death]. It’s not something that she [my wife] would like to discuss.” (#6*,* age 39)*.

The four participants who had children universally spoke about the challenges of how or when to approach the topic of mortality with their children.

*“She asked me*,* well*,* Mom*,* are you dying? I was like*,* “Oh*,* no” – but I was like*,* “How do I tell her ‘Yes’?” Like I know it’s going to happen*,* but… I want to do it soon because they gave me anywhere from three months to a year. So I want her to be like prepared mentally. I know she’s little*,* but I know I can do it.” (#3*,* age 23).*

Parents had different approaches to preparing their children for their potential death, some of which evolved over the course of their Bright IDEAS-YA participation. For example, one participant initially shared the belief that, if she pulled away from her children following her cancer diagnosis, they would mourn her loss less. By the time of her final session, this participant reported that her perspective had changed as a result of conversations with other members of her social network *“I have had a lot of opportunity to have a better relationship*,* even better than before*,* with my husband and my kids.” (#1*,* age 36).*

Another universal theme amongst parents was feelings of grief about the potential loss of future time with children:

*“Even just talking about*,* my daughter’s going to be starting college*,* actually even making it to her graduation*,* or my other daughter’s graduation in three years*,* something… I guess the biggest thing I look at it as being a problem is that I’m not there or wouldn’t be there to support those milestones.”(#6*,* age 39).*

Each of these four participants used Bright IDEAS-YA to consider the legacy they wanted to leave for their children, such as how they wanted their children to remember them, what sort of mementos they would like to leave for their children, and how they wanted to capitalize on time spent together.

### Social system

Participants’ reflections on their social systems made up a large proportion of session content. Participants’ social communities were described both as significant sources of support and as potential sources of distress. Participants’ social systems comprised family members (e.g. parents, in-laws, siblings) spouses or committed partners, children, friends, pets, coworkers, connections made through school, and connections related to an individual’s cancer experiences (e.g. support groups; mentorship relationships).

Participants reviewed how their social systems provided support through emotional and practical help. Loved ones were often described as helping participants manage their physical needs, such as remembering to eat despite limited hunger cues, or encouraging participants to reduce physiological demands on themselves. “*My cousin*,* who’s like my sister… she sometimes reminds me*,* too*,* just like*,* ‘Why are you still up cleaning?’ or ‘Can you go sit down?’ And I’m like*,* you’re right*,* I do deserve to sit down.” (#9*,* age 32).* There were many occasions in which participants described their social networks volunteering time, service, or resources to physically help them manage needs. *“When I first got diagnosed*,* we had people send us Uber Eats gift cards and stuff.” (#6*,* age 39).* Participants’ social systems were also framed as important in helping participants feel connected to their lives pre-cancer, and in offering distraction from ruminating on physical or emotional discomfort.

Conversely, members of participants’ social systems could be viewed as sources of anxiety or sadness. Many participants (*n* = 8, 67%) noted that changes in their capacity to participate in social engagements highlighted cancer-related challenges. One participant reflected on trying to schedule a meet-up with their sister and sister’s children, *“If I get up there and I exhaust myself*,* which she has two little kids*,* even sitting on a park bench having a conversation can be too much for me. So it’s not even like physical exhaustion*,* it can even be mental exhaustion… So it makes it difficult.” (#10*,* age 33).* Several participants (*n* = 4, 33%) found that their social networks were not always able to fully understand changes to their physical, emotional, and mental capacities, which felt isolating. Participants also noted that although others were typically sympathetic to their cancer diagnosis and treatment experiences, gestures of support could be unhelpful at times.

*“So many people know someone with cancer*,* and everybody’s story and journey is different. And everybody just insists on sharing their uncle*,* aunt*,* best friend’s story with somebody that has cancer. And I’ve had to stop people and be like*,* wait*,* does this story end well? Because*,* otherwise*,* I don’t want to hear it.” (#10*,* age 33).*

#### Talking to loved ones about cancer

For many participants (*n* = 7, 58%), an important early consideration was when and how to disclose their cancer to their social system: “*Right now I’ve told a lot of close friends about my condition. I’ve been having some trouble motivating myself to tell other people that aren’t as close to me*,* because I’ve been through the struggle of telling those really close to me. And I don’t really want to go through it again.” (#2*,* age 20).*

Participants had different wants and needs in terms of how they hoped their loved ones would dialogue about cancer: some participants (*n* = 2, 17%) described wanting loved ones to show optimism or humor, others (*n* = 2, 17%) expressed frustration with others’ optimism, describing that it felt painful when they themselves anticipated a negative outcome. Participants expressed how challenging it could be to sit with their loved ones’ sadness, although some also found value in sharing their feelings of grief.

“*When I talk to my mom and my brother*,* it’s like – we do get upset*,* but it’s not to the point where I’m like in tears*,* like we just turn it – we flip it right around and start joking about it. It’s just like*,* we just – I’d rather have that than be in tears with somebody.” (#3*,* age 23).*

Much as with explicit conversations about mortality, participants whose expectations of their prognoses differed from those of their loved ones often described this as affecting communication.

*“So just with my type of cancer*,* I’m wondering if it’s worth it to get a degree*,* because I won’t ever actually use it*,* and it’s also quite expensive for my parents… I think*,* if I talk to my parents*,* they definitely would tell me to stay in school*,* because they’re sort of in denial*,* I guess*,* and they say there’s a lot of cutting-edge treatments that will save me or something and that I should just continue life as normal.” (#2*,* age 20).*

It appeared meaningful for some participants to have others in their social community who had shared cancer experiences. Several participants (*n* = 4, 33%) reported finding this through support groups or by being put in touch with another person with the same kind of cancer. The participants who found this helpful often reflected that these connections reduced feelings of isolation and provided solidarity and context for their cancer journey. Other participants were hesitant to consider building a social network with others with cancer. These participants worried that having these connections would be demoralizing or saddening. One participant shared: *“Honestly*,* I don’t want to be in one of the groups with other people with cancer because I fall in love with people. I just love people. So if I was in one of those groups*,* and I lost somebody to cancer*,* I think I’d be really heartbroken.” (#10*,* age 33).*

### Work/Academics

All participants (*n* = 12, 100%) spoke about their occupational or educational experiences, and how these intersected with their cancer experience. Participants who were working or pursuing higher education frequently identified Bright IDEAS-YA goals and challenges related to cancer affecting occupational/academic functioning. Work/education-related challenges identified by participants included (1) requesting time off/medical leave; (2) approaching return to school/work; (3) adjusting to changes in anticipated educational/vocational trajectories; and (4) ensuring educational or workplace accommodations. Many participants reflected that it was difficult to determine what bureaucratic steps (e.g. paperwork) they needed to take. All participants who reflected on educational/vocational topics expressed that the uncertainty of their cancer trajectory made making educational/vocational decisions challenging.

*“I want to get back [to work] as soon as possible*,* but I don’t know what that’s gonna look like and when that’s gonna happen. So there’s a lot of unknowns there. I also don’t even know – the role I was in at work because I’m past FMLA at this point*,* I may not get that back. So I don’t even know what position I’m actually gonna get when I go back.” (#12*,* age 36).*

Of note, all five (42%) participants who were pursuing higher education took a leave of absence for at least one semester while on treatment. Participants who stopped attending school or going to their workplace often noted the impact of having decreased socialization opportunities.

#### Financial toxicity

Some participants (*n* = 8, 67%) discussed that constraints associated with the cost of treatment informed occupational decision-making. For people facing occupational uncertainty, this was a significant source of anxiety. As one participant described the dilemma, *“We got a call from our corporate bosses*,* and… what that means is really my job is only guaranteed for like 45 days*,* which is – like I told him*,* it’s extremely scary because I still have nine – at this time*,* I still have nine treatments and that’s over $1 million out of pocket.” (#9*,* age 32).* A subset of participants with employed spouses (*n* = 2, 17%) reported discussing changing insurance carriers to coverage offered by a spouse’s workplace to lessen the financial insecurity resulting from potentially losing their job.

### Mental health

Many participants appeared to be well-versed in mental health topics. Some participants reported historical diagnoses of, or symptoms associated with depression, anxiety, and attention-deficit/hyperactivity disorder (ADHD; n = 7, 58%) and a subset of these (*n* = 3, 25%) had previously sought mental health services (e.g. counseling, psychiatric medication management). A common theme amongst participants was that a history of mental health difficulties magnified the effects of cancer-related stressors.

*“I mean*,* I’ve always done this [overthinking]. Obviously*,* now*,* it’s the current issues with everything that goes along with the cancer diagnosis and unknown future and all that kind of stuff.” (#12*,* age 36)*.

One participant with ADHD noted that they paused their ADHD medication regimen when beginning cancer treatment, and that they felt ADHD compounded chemo-related brain fog. Some participants reported symptoms of mental health conditions (e.g. feeling down, perseverative worry about the future) without labelling these symptoms explicitly. Bright IDEAS-YA trainers commonly suggested individual or group counseling as a problem-solving strategy for the participant to consider. This suggestion was met with mixed reactions; some participants were skeptical that mental health symptoms could be reduced while cancer and mortality-anxiety, as a stressor, remained present.

*“I just can’t imagine how much it’ll [counseling] help. I mean*,* I guess – I mean*,* even just talking through things with you definitely helps*,* but there’s always some little things that no matter what*,* I still have cancer. You know*,* like that’s not gonna go away. “ (#5*,* age 24).*

Other participants who expressed hesitancy to access mental health services reported that they were unsure if they could afford care. Bright IDEAS-YA trainers supported participants in identifying means to access covered, low-cost, or pro bono mental health services.

### Spirituality

Several participants (*n* = 4, 33%) discussed religion and spirituality in the contexts of their cancer diagnosis and as it pertained to their own practice, identity, and experiences. Participants who had their own spiritual or religious practice described themselves as turning to spirituality and prayer for comfort when considering their cancer, treatment trajectory, and potential mortality. Those participants who endorsed religious practice reflected that their religion helped them find meaning in their cancer experiences: *“I believe in God. It’s almost like God intervened and was like*,* slow down for a minute. Take care of you. Take care of your health.” (#9*,* age 32).* Several participants (*n* = 3, 25%) felt that their positive belief and prayer could positively impact their cancer trajectory. The inverse of this, which was also endorsed by two participants, was that negative thinking would negatively impact their cancer and treatment outcomes. One participant expressed the feeling that their anticipation of death might affect their religious belief, which suggests awareness of proximity to end-of-life may affect end-of-life values *“I’d probably start believing in God a little more if I was gonna die.” (#4*,* age 20).*

Participants’ religious and spiritual orientation also appeared to impact their preference for turning to religious versus non-religious figures for support. Two participants who identified counseling as a potential solution to increase support considered counseling services through their place of worship.

*“My husband think I should probably spend a lot of time reading Bible or seeing him [participant’s pastor]*,* and maybe a lot of things that I’m worried will be answered while I’m reading the Bible. So my husband think I should do that rather than speak to some counseling.” (#1*,* age 36).*

Another participant was mistakenly referred to a chaplain when requesting mental health support, which they reported finding aversive due to their historical relationship with religious figures. *“I told my trial coordinator that I wanted to talk to somebody*,* but she sent over the… the chaplain. That’s what it was. And I mean*,* that’s okay*,* but I’m a pastor’s – I have two pastors in my family. And so I’ve had my fill of that” (#10*,* age 33).*

### COVID-19

Due to the timing of this RCT, participants frequently spoke about COVID-19 pandemic precautions and how quarantine affected their cancer experiences. Several participants (*n* = 6, 50%) reported heightened anxiety about their susceptibility to COVID-19, and the need for them and loved ones to be cautious about exposure, given their treatment-induced immune suppression. Some participants expressly appreciated friends and family exercising COVID-19 precautions around them (e.g. masking, practicing social distancing, vaccinations).

Because precautions changed visitation policy in clinical settings, several participants reported significant loneliness during inpatient stays and medical appointments, when they would have wanted to have more loved ones present for support.

### Meaning making

Participants described how cancer experiences informed their personal values. Participants who spoke to this theme (*n* = 7, 58%) expressed that intentionality and connection to loved ones had become more important to them through their cancer trajectory.

*“Even just those little moments*,* like I’m sure [deceased friend] would – and her family*,* you know – would do anything to have those little moments back*,* just sitting*,* watching TV. So I just try and think of even the boring moments as moments that I can at least be enjoying with my family.”(#5*,* age 24).*

One participant shared gratitude for how her cancer trajectory had informed her perspective: *“I feel like for people who die from accidents*,* like COVID*,* they never have that opportunity to say a lot of the things that they wanted to share with their loved ones. And I have that opportunity. So I feel like I have enough time.” (#1*,* age 36).*

One participant detailed, over several sessions, how her cancer diagnosis had informed her commitment to advocating for public education and access to genetic testing for cancer risk. During Bright IDEAS-YA, she helped several family members complete genetic testing and had planned to change careers to pursue advocacy in this field.

### Participation in Bright IDEAS-YA

Because all transcribed sessions were delivered as part of the Bright IDEAS-YA RCT, participants were asked to discuss aspects of their participation in the intervention. Participants often discussed Bright IDEAS-YA in positive terms, expressing excitement about their participation in the intervention; hopefulness about meeting challenges and goals; and pride when they were successful: *“I felt accomplished [completing Bright IDEAS-YA goal]. I felt like I didn’t waste away the whole day. So it was nice to get up and start into a routine and getting things done.” (#11*,* age 31).*

### Emotions

**Fig. 2:**
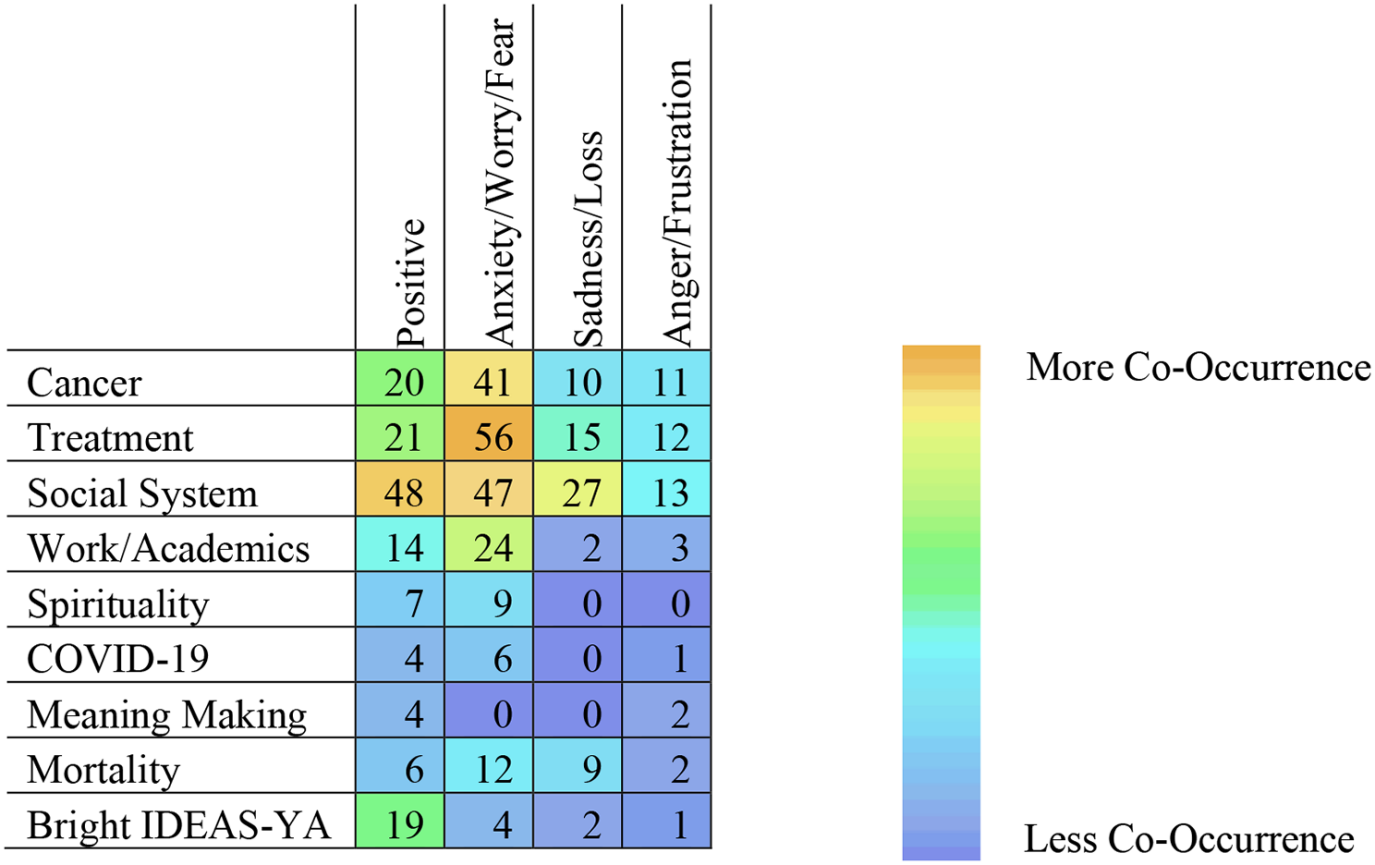
Frequency of Emotion Code Co-Occurrence with Identified Themes

A wide range of emotions were present as participants spoke about different aspects of their lives. Participants typically labelled emotions in-context, and coders categorized emotional valence into (1) positive emotions (e.g. happiness, contentedness, pride, gratitude); (2) anxiety, worry, and fear; (3) sadness, grief, and loss; and (4) anger and frustration. Anxiety-related and positive emotional experiences were coded most frequently, particularly when participants discussed cancer, treatment, and social systems. Participants often used emotion-terms, both positive and negative, when discussing their social systems. Participants labelled positive emotional experiences across themes, with the highest relative frequency of co-occurrent positive emotion codes – when compared to other codes – appearing in the Bright IDEAS-YA domain. Figure 2 displays a heat map detailing frequency of emotion code co-occurrences with codes correspondent to *Results* themes.

## Discussion

This study analyzed Bright IDEAS-YA session transcripts of YAs receiving cancer care at US cancer centers who went on to die or have unexpectedly rapid cancer progression during their participation in the Bright IDEAS-YA RCT. Emergent themes illustrated that these YAs faced challenges throughout their cancer care, similar to other YAs with cancer who have not been explicitly identified as being at end-of-life [11, 12]. Participant YAs’ personal values and life situations informed how they addressed these challenges.

Participants frequently identified that the time from symptom identification to cancer diagnosis to treatment felt rapid, which was unexpected, given literature speaking to delays in diagnosis for YAs [24]. This theme may reflect participants’ subjective interpretations, based on feelings while in active treatment, or could be reflective of these YAs’ cancer being more advanced when they initially presented for medical care.

Patient-provider communication was important in facilitating participants’ linkage to resources, and, in contrast, ineffective patient-provider communication inhibited patient outreach. Participants’ receptivity to resource-linkage increased when provider recommendations and resources were individually- and developmentally-tailored, mirroring recommendations from the literature [25]. Findings on participants’ experiences in social contexts – with healthcare providers, family, friends, and children – exemplified the nuance of YAs’ social networks, which could be both sources of support and distress. This is in alignment with literature speaking to the importance of understanding YAs’ social networks [26–28]. Challenges in communicating about their diagnosis and prognosis seemed particularly salient for participants who were parents.

As this study took place beginning in 2021, many participants reported that COVID-19 exacerbated cancer-related educational/vocational disruptions, and several participants described loneliness related to pandemic precautions. Participants used Bright IDEAS-YA to address concerns around financial wellbeing, which emphasizes the importance of recent attention paid to increasing patients’ awareness of vocational planning and potential financial toxicity at the time of diagnosis [29–31].

YA cancer patients are also at high risk of experiencing significant distress [32]. Many participants identified themes corresponding to high emotional valence and frequently spoke about the intersection of cancer and their mental health needs. Many participants valued linkage to mental health resources, but some expressed hesitancy about accessing services. The literature shows that YA cancer survivors experience significant mental health needs, but most do not obtain professional mental health services [33]. Lessons gleaned from our study indicate that it is important that (1) mental health service referrals be tailored to YAs’ individual values (e.g. pastoral vs. secular mental health services); (2) patients are educated about means of accessing financially feasible mental health services and resources (e.g. covered, low-cost, pro bono); and (3) mental health services should be routinely offered as part of comprehensive care as a strategy to reduce stigma around their use. Such strategies may help decrease the gap between mental health needs and care delivery.

This analysis demonstrated that this sample of YAs with cancer, who were retrospectively identified as being at end-of-life or whose condition declined unexpectedly rapidly, had lived experiences that mapped closely onto those of other YAs with cancer, outside of an end-of-life context. This is not entirely surprising, as these YAs were recruited into this study with the expectation of living for at least six months or longer, and ultimately, only a minority of these patients died within a few months of their Bright IDEAS-YA sessions. However, the data suggest that patients are aware of and willing to talk about end-of-life issues in the context of serious potentially life-ending illness, even if death is not apparently imminent. On the other hand, some patients even with a short time to live may not bring up these issues.

In recent decades, there has been growing movement to systematically introduce advanced care planning and/or end-of-life discussions for all YA patients diagnosed with life-threatening illness, in order to improve healthcare providers’ ability to meet patients’ wishes and values, and to reduce the possibility that patients whose conditions decline rapidly fall into service gaps in end-of-life care [34–36]. This analysis supports that, in many ways, these YA patients at end-of-life were not distinct from other cohorts of YA patients. For healthcare providers seeking to meet these patients’ needs, it is imperative that these patients are understood both as young adults, seeking to live their lives in the context of cancer, and as people who may be facing life-limiting illness. In both of these contexts, providers should seek to understand their patients’ priorities, values, and goals. Supportive services can help YAs make decisions that align with their priorities and values, regardless of the time they have to live. These findings also align with global efforts to understand death literacy, defined as a multidimensional construct including the knowledge, skills, and community capacity to identify and act on end-of-life care options [37]. Further work is needed to quantify and understand death literacy in young adults with cancer, which was beyond the scope of the current study.

### Limitations

The post-hoc identification of this sample is both a strength and a limitation. Due to the unpredictable nature of cancer trajectories, the authors drew a sample of YAs where neither participants nor study staff knew their proximity to death or unexpected medical decline at time of study enrollment. Because of how the larger psychosocial intervention RCT was designed, these individuals’ study participation has offered a unique opportunity to examine psychosocial experiences of YAs with cancer during unanticipated end-of-life or decline. Although bias may occur in accidental sampling of this kind, it is possible that it has led to greater ecological validity. With this said, it must also be acknowledged that participants’ very participation in this intervention could have impacted the themes that arose in this analysis. Bright IDEAS-YA is designed to be patient-led and facilitate patients’ own goals and priorities, but it must still be considered that this dataset was not derived from naturalistic observation.

Due to the unique means by which this sample was identified, there are also limitations with regard to its representativeness. This sample was predominantly women, and majority white. In that individuals’ experiences with end-of-life vary meaningfully between cultures and across the globe – and all participants within this study sample were YAs receiving cancer care at US institutions – caution should be taken in overgeneralizing this data. Though authors continued to sample participants through to thematic saturation, it is possible that, even within the larger Bright IDEAS-YA study population, different themes may have emerged, should a different subset of RCT participants have approached end-of-life unexpectedly.

As with any qualitative research, there is risk of subjective bias in data analysis and interpretation. The authors sought to mitigate this by employing triangulation in methods and ensuring intercoder consensus throughout analysis [21, 22]. The literature will benefit from (1) further qualitative investigations into these patients’ lived experiences to broaden our understanding, and (2) quantitative data speaking to patients’ needs and psychosocial wellbeing prior to unanticipated death.

## Conclusion

By better understanding the experiences of YA cancer patients who go on to unexpectedly approach end-of-life or experience rapid health decline, we can provide more effective patient-centered clinical services prior to and during this part of their care trajectory. Our participants expressed uncertainty in navigating many cancer-related challenges, similar to other YAs diagnosed with cancer. Some participants readily approached the topic of their own mortality, and others did not. These YAs’ experiences were idiosyncratic and complex, as well as highly emotionally valent. Overall, the importance of patient-tailored care – informed by regular check-ins with patients’ present physical capacity and personal priorities – was evident throughout themes. Healthcare settings applying systematized advanced care planning/end-of-life procedures for any patient with life-threatening illness will help center the values and goals of patients whose health trajectories rapidly decline [35, 36].

## Electronic supplementary material

Below is the link to the electronic supplementary material.

Supplementary Material 1

## Data Availability

The data are available from the corresponding author upon request.

## Footnotes

1 The Bright IDEAS-YA model is stepwise and leads participants through problem-solving steps via the IDEAS acronym: “I”= “Identify the problem”; “D”=“Determine your options”; “E”=“Evaluate options and choose the best”; “A” = “Act”; and “S”=“See if it worked” [18].
