# Supplementary material for: “There’s a lot of unknowns”: a thematic analysis of the experiences of young adults with cancer who died during a psychosocial intervention trial
Source: BMC Palliat Care. 2025 Apr 9;24:98. doi: 10.1186/s12904-025-01725-2 (PMC11980252; doi:10.1186/s12904-025-01725-2)
Supplement: Supplementary file 1 — Supplementary Material 1 [file 12904_2025_1725_MOESM1_ESM.docx]

**BrightIDEAS Session Codebook**

**Contents**

[Cancer 3](#_Toc138024069)

[Dx: Learning about Dx 3](#_Toc138024070)

[Dx: Symptoms 3](#_Toc138024071)

[Dx: Expectations 3](#_Toc138024072)

[Cancer Treatment 3](#_Toc138024073)

[Tx: Side-effects 3](#_Toc138024074)

[Tx: Schedule 4](#_Toc138024075)

[Tx: Palliative 4](#_Toc138024076)

[Tx: Provider Relationship 4](#_Toc138024077)

[Clinical Setting 4](#_Toc138024078)

[Setting: Emergency Room 4](#_Toc138024079)

[Setting: Inpatient Hospitalization 4](#_Toc138024080)

[COVID-19 5](#_Toc138024081)

[COVID: Adaptations 5](#_Toc138024082)

[COVID: Challenges 5](#_Toc138024083)

[COVID: Vaccine 5](#_Toc138024084)

[Living Situation 5](#_Toc138024085)

[Living Situation: Moving 5](#_Toc138024086)

[Living Situation: Insecurity 5](#_Toc138024087)

[Social System 6](#_Toc138024088)

[Social System: Family 6](#_Toc138024089)

[Social System: Significant Other 6](#_Toc138024090)

[Social System: Friend 6](#_Toc138024091)

[Social System: Classmate/Coworker 6](#_Toc138024092)

[Social System: Cancer Connections 6](#_Toc138024093)

[Work or Education 7](#_Toc138024094)

[Work: Future 7](#_Toc138024095)

[Work: Adjustments 7](#_Toc138024096)

[Religion/Spirituality 7](#_Toc138024097)

[Mortality 7](#_Toc138024098)

[Mental Health 7](#_Toc138024099)

[Mental Health: Endorses 7](#_Toc138024100)

[Mental Health: Services 8](#_Toc138024101)

[Emotions 8](#_Toc138024102)

[Emotion: Happiness/ Contentedness 8](#_Toc138024103)

[Emotion: Sadness 8](#_Toc138024104)

[Emotion: Anger 8](#_Toc138024105)

[Emotion: Anxiety/Fear 8](#_Toc138024106)

[Problem-Solving Style 8](#_Toc138024107)

[BrightIDEAS Sessions 9](#_Toc138024108)

[Problems Identified 9](#_Toc138024109)

[Problems: Thinking about Cancer 9](#_Toc138024110)

[Problems: Social Challenges 9](#_Toc138024111)

[Problems: Mental/ Emotional Health 9](#_Toc138024112)

[Problems: Physiological 9](#_Toc138024113)

[Problems: External Stressors 10](#_Toc138024114)

[Solutions Identified 10](#_Toc138024115)

[Solutions: Acceptance 10](#_Toc138024116)

[Solutions: External Change 10](#_Toc138024117)

[Solutions: Internal Change 10](#_Toc138024118)

[Pros 10](#_Toc138024119)

[Cons 11](#_Toc138024120)

[Cons: Unknown Helpfulness 11](#_Toc138024121)

[Quotes 11](#_Toc138024122)

**Instructions**

Each code in the codebook below has a description to guide code’s application. ***As many codes as apply can be assigned to a single excerpt.*** Green (level 1 header) denotes parent codes. Blue (level 2 header) denotes child codes and purple (level 3 header) denotes grandchild codes.

# Cancer

*Description:*

Participant describes any aspect of their cancer diagnosis.

## Dx: Learning about Dx

*Description:*

Child code for **Cancer**. Participant describes when they learn about their cancer diagnosis. Includes participant initially learning about their cancer and instances where participant learns new diagnostic information (e.g. new developments in cancer trajectory, new cancer diagnoses, etc.).

## Dx: Symptoms

*Description:*

Child code for **Cancer.** Participant describes specific symptoms associated with their cancer.

## Dx: Expectations

*Description:*

Child code for **Cancer**. Participant describes expectations about their cancer diagnosis and/or the trajectory of their cancer. May be positively- or negatively-valanced. May merit co-coding with **Tx: Expectations**, **Mortality**, and/or **Problems: Thinking about Cancer**.

# Cancer Treatment

*Description:*

Participant describes aspects of their oncology care. Can include any aspect of treatment, including chemo, radiation, surgery etc., as well as monitoring procedures (e.g. CT scans, x-rays, etc.). When described treatment is explicitly palliative in nature, include child code **Tx: Palliative**.

## Tx: Side-effects

*Description:*

Child code for **Cancer Treatment**. Participant describes side-effects associated with their cancer treatment (i.e. chemo, radiation, etc.)

## Tx: Schedule

*Description:*

Child code for **Cancer Treatment**. Participant describes aspects of their treatment schedule (e.g. “I do chemo on Wednesday”). May include challenges encountered associated with their treatment scheduling.

## Tx: Palliative

*Description:*

Child code for **Cancer Treatment**. Participant describes palliative care explicitly. DO NOT use this code if a component of treatment could be palliative, but is not explicitly described as palliative by participant (e.g. participant is put on high dose of morphine).

## Tx: Provider Relationship

*Description:*

Child code for **Cancer Treatment**. Participant describes aspects of their relationship with their oncology provider. May include examples of collaborative decision-making, challenges with providers, etc.

# Clinical Setting

*Description:*

Participant explicitly describes a clinical setting. Do not use code if a clinical setting is implied but not explicitly described (e.g. “I had chemo on Wednesday”).

## Setting: Emergency Room

*Description:*

Child code for **Clinical Setting**. Use when participant describes going to the ER/ED. Can be at any point in treatment (pre-dx, post-dx).

## Setting: Inpatient Hospitalization

*Description:*

Child code for **Clinical Setting**. Use when participant explicitly describes inpatient hospitalization experiences.

# COVID-19

*Description:*

Participant references the COVID-19 pandemic.

## COVID: Adaptations

*Description:*

Child code for **COVID-19**. Participant describes adaptations to daily life associated with the pandemic or pandemic-related precautions.

## COVID: Challenges

*Description:*

Child code for **COVID-19**. Participant describes challenges associated with the pandemic or pandemic-related precautions.

## COVID: Vaccine

*Description:*

Child code for **COVID-19**. Participant discusses the COVID-19 vaccine and/or thoughts about the vaccine.

# Living Situation

*Description:*

Participant describes their home or living environment.

## Living Situation: Moving

*Description:*

Child code for **Living Situation**. Participant describes moving during their cancer diagnosis and/or treatment. May include temporary moves (e.g. staying with family who lives close to hospital during radiation treatment). DO NOT include excerpts where participants describe moving prior to their cancer experiences (e.g. “I moved to this state when I was a kid”).

## Living Situation: Insecurity

*Description:*

Child code for **Living Situation**. Participant describes a living situation in which they are experiencing housing insecurity. This includes situations where participants have had to move due to financial stressors, are having trouble paying rent, etc.

# Social System

*Description:*

Participant describes other individuals and/or communities in their lives. Includes references to others in all variety of contexts.

## Social System: Family

*Description:*

Child code for **Social System**. Participant references family member(s).

## Social System: Romantic Interest

*Description:*

Child code for **Social System**. Participant references their significant other or a potential dating partner. Can include recent exes, if the individual in question has had a significant other role around the time of participant’s cancer diagnosis or treatment. When significant other is spouse, co-code with **Social System: Family.**

## Social System: Friend

*Description:*

Child code for **Social System**. Participant references friend(s).

## Social System: Classmate/Coworker

*Description:*

Child code for **Social System**. Participant references acquaintances they are exclusively involved with through work or school. If a classmate or coworker is close enough with the participant to be considered a friend, code **Social System: Friend** instead.

## Social System: Cancer Connections

*Description:*

Child code for **Social System**. Participant references relationships made through their cancer experience. May include members of cancer support groups, online cancer forums, etc.

## Social System: Online Only

*Description:*

Child code for **Social System**. Participant references social connections made online with individuals that they have not met in real life. Can be through forums, dating apps, etc. Do not code when participants are discussing online means of connecting with people they have met in real life.

## Social System: Pets

*Description:*

Child code for **Social System**. Participant references their pets or pets that they feel connected to (e.g. family pets, significant others’ pets).

# Work or Education

*Description:*

Participant describes their job, education, vocational training activities. Can include present, past, or future-oriented statements.

## Work: Future

*Description:*

Child code for **Work or Education**. Participant describes hopes or plans for their future education/career.

## Work: Adjustments

*Description:*

Child code for **Work/Education**. Participant describes any adjustments made to their work or education associated with their cancer or treatment. May include accommodations in workplace/school, may include taking leave from work or school.

# Religion/Spirituality

*Description:*

Participant describes their relationship with religion or spirituality, and/or their lack thereof.

# Mortality

*Description:*

Participant discusses content related to death, could be in reference to thoughts of their own mortality or others’.

# Mental Health

*Description:*

Participant describes content related to their mental health.

## Mental Health: Endorses

*Description:*

Child code for **Mental Health**. Participant explicitly endorses having a mental health condition. (e.g. “My depression has been really bad lately”). DO NOT code symptoms that may be associated with a mental health condition (e.g. “I’ve just had low motivation”) UNLESS the participant explicitly describes them as connected to mental health condition (e.g. “My depression has really made motivation hard.”)

## Mental Health: Services

*Description:*

Child code for **Mental Health**. Participant describes engagement with present or past mental health services outside of BrightIDEAS.

# Emotions

*Description:*

Participant speaks to their emotional experience. If it is unclear which child code that should be used to categorize an emotion (e.g. “confused”, “numbness”, “I don’t feel anything”, or “I don’t know how I feel”), only code with **Emotions** parent code.

## Emotion: Happiness/ Contentedness

*Description:*

Child code for **Emotions**. Participant describes an emotional experience related to happiness or contentedness (e.g. “happy”, “fine”, “not too bad”, “grateful”).

## Emotion: Sadness

*Description:*

Child code for **Emotions**. Participant describes an emotional experience related to sadness, grief, loneliness (e.g. “sad”, “devasted”, “lonely”).

## Emotion: Anger

*Description:*

Child code for **Emotions**. Participant describes an emotional experience related to anger or frustration (e.g. “angry”, “annoyed”).

## Emotion: Anxiety/Fear

*Description:*

Child code for **Emotions**. Participant describes an emotional experience related to anxiety, or fear (e.g. “worry”, “afraid”, “anxious”).

# Problem-Solving Style

*Description:*

Participant describes content related to their style of solving problems.

# BrightIDEAS Sessions

*Description:*

Participant discusses BrightIDEAS sessions (e.g. their response to BrightIDEAS content, their engagement with BrightIDEAS model outside of session, scheduling with BrightIDEAS clinician). Do not code for excerpts in which participant is simply moving through the BrightIDEAS model (e.g. participant identifies pros and cons of a proposed solution).

## BrightIDEAS: Thought Restructuring

*Description:*

Child code for **BrightIDEAS Sessions**. Participant is led through thought restructuring exercise as part of the BrightIDEAS model.

# Problems Identified

*Description:*

In the context of the BrightIDEAS model, participant identifies a problem that they would like to work on.

## Problems: Thinking about Cancer

*Description:*

Child code for **Identified Problems**. Participant identifies thoughts about cancer or treatment as a problem. This can include rumination on cancer or treatment, anticipation about their cancer/treatment trajectory, and concerns about negative cancer-related outcomes.

## Problems: Social Challenges

*Description:*

Child code for **Identified Problems**. Participant identifies some aspect of their social experience as a problem. May include feelings of isolation, interpersonal discord, or concerns about the impact of their cancer on their social relationships (e.g. “How do I tell others about my cancer?”).

## Problems: Mental/ Emotional Health

*Description:*

Child code for **Identified Problems.** Participant identifies concern related to their mental and emotional health. This code is used for mental health/emotional concerns that are not explicitly related to cancer thoughts, although can be related to their cancer experiences (e.g. “I’ve noticed that I haven’t really had as much motivation since chemo”.) Includes concerns related to procrastination.

## Problems: Physiological

*Description:*

Child code for **Identified Problems.** Participant identifies a concern related to their body. May be related to cancer and cancer experiences (e.g. “Since chemo, I am so fatigued”) or unrelated (e.g. “I gained a lot of weight after my pregnancy”). Include cognitive changes associated with chemotherapy (e.g. “because of chemo-fog, I can’t pay attention”).

## Problems: External Stressors

*Description:*

Child code for **Identified Problems**. Participant identifies an external stressor that is not interpersonal. May include the environment not sufficiently adapting to a participant’s needs, housing insecurity, COVID-related stress, etc. If an external stressor maps onto another code, co-code with that code.

## Problems: Maximizing Time

*Description:*

Child code for **Identified Problems**. Participant identifies a problem or goal related to spending time left meaningfully.

# Solutions Identified

*Description:*

In the context of the BrightIDEAS model, participant reflects on or identifies potential solutions to their problems.

## Solutions: Acceptance

*Description:*

Child code for **Identified Solutions**. Potential solution takes an acceptance-based approach. (e.g. “Tell myself that it’s okay I feel this way”).

## Solutions: External Change

*Description:*

Child code for **Identified Solutions**. Potential solution incorporates participant changing external circumstances (e.g. “I will ask my doctor for more information about this”, “I will plan to work out 3x per week”).

## Solutions: Internal Change

*Description:*

Child code for **Identified Solutions**. Potential solution incorporates participant changing their perspective on something or “cognitive reframing” (e.g. “Based on the evidence, I’ll remind myself my cancer isn’t usually dangerous”).

# Pros

*Description:*

In the context of the BrightIDEAS model, participant discusses pros associated with a potential solution.

# Cons

*Description:*

In the context of the BrightIDEAS model, participant discusses cons associated with a potential solution.

## Cons: Unknown Helpfulness

*Description:*

Child code for **Cons**. A participant conveys being unsure if a potential solution will be helpful when identifying cons associated with a potential solution.

# Quotes

*Description:*

Use to highlight meaningful or striking quotes.
